# Effects of a selectively bred novelty-seeking phenotype on the motivation to take cocaine in male and female rats

**DOI:** 10.1186/2042-6410-2-3

**Published:** 2011-03-11

**Authors:** Jennifer A Cummings, Brooke A Gowl, Christel Westenbroek, Sarah M Clinton, Huda Akil, Jill B Becker

**Affiliations:** 1 Molecular and Behavioral Neuroscience Institute, University of Michigan, Ann Arbor, MI 48109, USA; 2 Department of Psychiatry, University of Michigan, Ann Arbor, MI 48109, USA; 3 Department of Psychology, University of Michigan, Ann Arbor, MI 48109, USA; 4 Office of Research and Grants, Ohio University College of Osteopathic Medicine, Athens, OH 45701, USA

## Abstract

**Background:**

Gender and enhanced novelty reactivity can predispose certain individuals to drug abuse. Previous research in male and female rats selectively bred for high or low locomotor reactivity to novelty found that bred High Responders (bHRs) acquire cocaine self-administration more rapidly than bred Low Responders (bLRs) and that bHR females in particular self-administered more cocaine than the other groups. The experiments presented here aimed to determine whether an individual's sex and behavioral phenotype interact to affect motivation to take cocaine.

**Methods:**

We examined motivation for taking cocaine in two experiments using a range of doses on a progressive ratio (PR) schedule of responding in bHR or bLR males and females. Additionally, we included a measure of continuing to respond in the absence of reinforcement, a feature of addiction that has been recently incorporated into tests of animal models on the basis of the criteria for substance use disorder in the *Diagnostic and Statistical Manual of Mental Disorders, Fourth Edition*. Statistical analyses were performed using PASW Statistics 18.0 software. Data were analyzed using repeated-measures analysis of variance followed by a Bonferroni correction *post hoc *test when applicable.

**Results:**

We found sex differences as well as effects of novelty reactivity on the motivation to self-administer cocaine. Specifically, females demonstrated higher breaking points on the PR schedule compared with males, regardless of phenotype, and bHR males and females exhibited higher motivation than bLR animals at a number of the doses studied.

**Conclusions:**

An individual's sex continues to be a predisposing factor with respect to drug abuse liability and can be compounded by additional individual differences such as reactivity to novelty.

## Background

Drug abuse vulnerability can vary with an individual's sex, hormone status and personality traits (that is, novelty reactivity), among other factors. In humans, personality traits such as novelty-seeking, reactivity to novelty, sensation-seeking and impulsivity contribute to an enhanced likelihood of substance abuse [1-4]. People who exhibit a heightened response to novelty use more drugs compared with those with an attenuated novelty reactivity response. Thus, novelty responsivity is considered to be a predictor of drug abuse liability.

Analogously to humans, individual behavioral traits in rats influence psychostimulant self-administration [5-8]. Indeed, animals that exhibit high novelty-induced locomotor behavior (High Responders (HRs)) have increased sensitivity to the reinforcing effects of drugs compared with Low Responders (LRs) [7]. Numerous studies have used the HR-LR model to demonstrate the relationship between novelty-induced locomotor activity, drug taking and other risk-taking behaviors [7,9].

A line of rats has been selectively bred on the basis of their locomotor behavior in a novel, mildly stressful environment, thus producing bred High Responder (bHR) and bred Low Responder (bLR) rats [10]. This selectively bred line of animals shares many characteristics with outbred animals that are commercially purchased and subsequently screened and classified to be HRs or LRs [7,11]. The bHR rats exhibit enhanced acquisition of cocaine self-administration relative to bLR rats [5] as well as increased impulsivity (for example, reduced behavioral inhibition or increased responding on a task requiring inhibition compared with bLR rats) [12]. The bHR rats also exhibit an enhanced corticosterone response to stress and higher levels of glucocorticoid receptor mRNA expression in the hippocampus relative to bLR rats [13]. The experiments using the bHR and bLR rat lines demonstrate that the phenotypes are strongly genetically driven and highly predictable from one generation to the next [10]. The phenotypic differences in reactivity to novelty have become more pronounced and less variable with subsequent generations of breeding, such that phenotypic classification of animals can be predicted with >95% certainty. Selective breeding has also amplified many of the differences seen in outbred animals and has revealed additional components of the phenotype that appear to be significant with respect to drug abuse liability [12].

An additional factor that puts individuals at increased risk for drug abuse is whether the individual is female or male. Women begin using cocaine at earlier ages, progress through the stages of addiction more quickly, enter rehabilitation facilities after a shorter period of use and have shorter periods of abstinence compared with men [14]. Women also report a stronger dependence on cocaine and greater cue-induced craving compared with men [15]. Hormones have been indicated in some of the effects, as women also report greater pleasure from smoked cocaine when their estradiol levels are elevated during their menstrual cycle [16,17].

The preclinical literature supports the idea that there is a biological basis for, and a hormonal modulation of, effects of sex and hormones on drug abuse liability. Female rats demonstrate greater behavioral sensitization to cocaine after repeated cocaine treatment compared with males [18,19] and acquire cocaine self-administration more readily than males [20-24]. Estradiol enhances the reinforcing effects of cocaine, as female rats work harder to obtain cocaine during estrus when estradiol is high or when ovariectomized (OVX) rats are given estradiol [22,25,26], and they prefer higher doses of cocaine when in estrus compared with the other phases of the cycle [27]. Females also work harder on a progressive ratio (PR) schedule when in estrus or after estradiol treatment of OVX rats [25,28].

Recent data from our laboratory demonstrated that bHR male and female rats acquire cocaine self-administration behavior significantly faster than bLR male and female rats and also that bHR female rats ingest greater amounts of cocaine compared with bHR males as well as bLR males and females [5]. On the basis of these findings, we wanted to know whether increased motivation for the drug was the cause of bHR females' greater drug intake. Therefore, the current study sought to determine the impact of sex and the bHR and bLR behavioral phenotypes on the motivation to self-administer cocaine using a PR schedule with a range of doses of cocaine, with the inclusion of a test to examine the animals' responses to cocaine availability in the absence of reinforcement.

## Materials and methods

### Animals

Adult bHR and bLR male and female Sprague-Dawley rats (~100 days old) were acquired from the Akil Laboratory (University of Michigan, Ann Arbor, MI, USA) in-house breeding colony, where lines are maintained. Animals from the 18th and 19th generations were used for Experiment 1, and animals from the 21st, 23rd and 24th generations were used for Experiment 2. A description of the breeding strategy and initial behavioral characterization of the bHR and bLR lines was published previously [10]. Male and female rats were housed in a 14-hour light:10-hour dark cycle (lights on at 7:00 AM). Food and water were available *ad libitum*, and all experiments were conducted in accordance with the National Institutes of Health guidelines on laboratory animal use and care using a protocol approved by the University of Michigan Committee on Use and Care of Animals.

### Surgical procedures

Approximately 5 days following transfer from the breeding colony room, rats underwent implantation of indwelling intravenous jugular catheters connected to a back port. Jugular catheter construction and implantation were performed using previously described procedures [29]. In short, catheters were constructed by gluing Silastic tubing (0.51 mm inner diameter × 0.94 mm outer diameter, Dow Corning, Midland, MI, USA) to an external guide cannula (22-G guide cannula; Plastics One, Roanoke, VA, USA) using cranioplastic cement. A polypropylene mesh was secured to the bottom of the cannula using the same cement. Rats received an injection of buprenorphine (0.02 mg/kg subcutaneous injection) 30 minutes before they were anesthetized with isoflurane (5% isoflurane in oxygen). The free end of the Silastic tubing of the catheter apparatus was inserted into the right jugular vein of the animal and secured using 4-0 silk sutures around the tubing and the venous tissue. The catheter port exited dorsally from the animal. After successful implantation, the animal's catheter was flushed with 0.2 mL each of heparin (30 U/mL in 0.9% sterile saline) and gentamicin (3 mg/kg) to prevent clotting and infection, respectively. A dummy stylet was then inserted into the port opening. Two days after surgery the catheters were flushed with 0.2 mL of heparin (30 U/mL in 0.9% sterile saline) and gentamicin (3 mg/kg) and with gentamicin every day after that. Prior to the beginning of each cocaine self-administration session, the catheters were flushed with 0.1 mL of sterile saline and, following the rats' cocaine self-administration, the catheters were flushed with gentamicin (3 mg/kg). Female estrous cycle was monitored via daily vaginal lavage and microscopic examination of vaginal cells collected immediately following the cocaine self-administration session. Catheter patency was checked weekly using a solution of Pentothal (thiopental sodium, 15 mg/mL concentration; range, 0.08 to 0.20 mL) in sterile water.

### Cocaine self-administration apparatus

Cocaine self-administration was performed in standard operant chambers (Med Associates, Inc., Georgia, VT, USA), during which time animals could poke their noses ("nose poke") into the active hole for cocaine or into an inactive hole, which resulted in no consequences. Rats were connected to the infusion syringe via a swivel mounted to a counterbalanced arm, which allowed animals to move freely in the testing environment.

### Experiment 1: cocaine self-administration protocol

#### Fixed ratio training (week 1)

One week following catheter implantation animals were allowed to nose poke to self-administer cocaine using a fixed-ratio 1 (FR1) schedule of reinforcement (that is, one nose poke in the active hole resulted in receipt of one intravenous infusion of cocaine) for 3 hours daily for 5 days at a cocaine dose of 0.5 mg/kg/infusion with a maximum of 15 infusions possible per day. At the start of the test a house light was turned on, indicating drug availability. During FR1 testing, each active hole nose poke resulted in a 50-μL infusion of cocaine HCl delivered over 2.8 seconds accompanied by a stimulus light in the active hole. Each infusion during this portion of the test was followed by a 5-second timeout period, during which time nose pokes were recorded but had no consequences. The dose (0.5 mg/kg/infusion) was chosen because it is one that has been shown to result in fairly rapid acquisition of the drug-taking behavior [23]. Twelve animals began the experiment in each treatment group. Animals that reached the criteria of 15 infusions on the FR1 schedule were moved to the FR2 schedule (two active hole nose pokes resulted in one infusion). Only animals that achieved 15 infusions on the FR2 schedule continued to the PR schedule. The total number of animals to complete the study comprised 11 bHR females, 9 bLR females, 8 bHR males and 5 bLR males. Animals that failed to acquire self-administration behavior (that is, those that did not receive 15 infusions on the FR1 and FR2 schedules, comprising two bLR females, one bHR male and five bLR males) or animals that lost patency during the experiment (one bHR female, one bLR female, three bHR males and two bLR males) were removed from the study. The low number of bLR males acquiring drug-taking behavior is in agreement with our previously published data [5].

#### Progressive ratio testing (weeks 2 to 4)

Animals had 2 days off and then began daily cocaine self-administration sessions on a PR schedule for 5 days/week for 3 weeks using cocaine doses of 0.3 mg/kg/infusion, 0.4 mg/kg/infusion and 0.5 mg/kg/infusion, respectively. While the dose range was narrow, significantly different effects have been found with respect to drug taking [24,26]. Each dose was given for 1 week, and doses were counterbalanced among animals and across groups using a Latin square design. We utilized a 6-hour PR schedule of reinforcement that escalated through an exponential series, namely, 1, 3, 6, 9, 12, 17, 24, 32, 42, 56, 73, 95, 124, 161, 208 required nose pokes per infusion and so on, adapted from Richardson and Roberts [30]. A higher breaking point (BP) is an indication that the animal is more motivated to get the cocaine, as they are willing to work harder to obtain an infusion of the drug. The number of infusions, nose pokes in the active hole, nose pokes in the inactive hole and last completed ratio within 1 hour of the last infusion (BP) were recorded. The session terminated after 6 hours or if 1 hour elapsed without an infusion.

### Experiment 2: cocaine self-administration protocol

#### Fixed ratio training (week 1 and days 1 and 2 of week 2)

One week following catheter implantation animals were allowed to nose poke to self-administer cocaine using the FR1 and FR2 schedules for 3 hours daily for 7 days (5 days on, 2 days off, 2 days on) at a cocaine dose of 0.4 mg/kg/infusion with the maximum number of infusions possible being 15. Animals that reached the criteria of 15 infusions on the FR1 schedule were moved to the FR2 schedule. Animals remained on the FR2 schedule until PR training and were moved to the next phase of the experiment only if they achieved 15 infusions on the FR2 schedule. After excluding animals that did not acquire the behavior (one bHR male and two bLR males) or those that had catheters that were not patent (one bHR female, two bLR females, two bHR males and two bLR males), the final group numbers were 11 bHR females, 10 bLR females, 9 bHR males and 8 bLR males.

#### Progressive ratio training (days 3 to 5 of week 2)

Animals were then given 3 days of experience on the PR schedule at a cocaine dose of 0.4 mg/kg/infusion. We used the same PR schedule used in Experiment 1. The number of infusions, nose pokes in the active hole, nose pokes in the inactive hole and last completed ratio within 1 hour (BP) were noted.

#### Progressive ratio testing (weeks 3 to 5)

Animals had 2 days off and then began daily cocaine self-administration sessions on a PR schedule of 5 days/week for 3 weeks using cocaine doses of 0.25 mg/kg/infusion, 0.5 mg/kg/infusion and 0.75 mg/kg/infusion, respectively. Each dose was given for 1 week, and doses were counterbalanced as described above using the same procedures described for Experiment 1.

#### Fixed ratio 5 (week 6)

For 4 days animals were put on an FR5 schedule for two 40-minute periods per day (separated by a 15-minute drug nonavailability period) during which they could self-administer cocaine (0.4 mg/kg/infusion) on a FR5 schedule (each infusion was followed by a 40-second timeout) with a maximum of 50 infusions/period (11 bHR females, 8 bLR females, 9 bHR males and 5 bLR males; numbers were reduced compared to week 2 because of loss of patency). Drug availability was signaled by illumination of the house light, which was turned off when the drug was unavailable. The second FR5 drug availability session was used to prevent extinction of the drug-taking behavior. The number of infusions and the number of active and inactive pokes (during drug-available and nonavailable sessions) were recorded, as well as the number of inefficient pokes (pokes during the 40-second timeout), which did not count toward receiving an infusion.

### Statistics

Statistical analyses were performed using PASW Statistics 18.0 software (SPSS, Inc., Somers, NY, USA). FR1, FR2 and PR data were analyzed using repeated-measures analysis of variance (ANOVA) with dose and day as within-subject variables and phenotype (bLR or bHR) and sex (male or female) as between-subject variables, followed by a Bonferroni correction *post hoc *test when applicable. Data obtained with the FR5 reinforcement paradigm were analyzed separately for the drug availability and nonavailability periods using repeated-measures ANOVA with day as the within-subject variable and phenotype and sex as between-subject variables, followed by a Bonferroni correction *post hoc *test when applicable. For the drug-available sessions, the number of nose pokes in the active and inactive holes, number of infusions and number of ineffective nose pokes in the active hole were analyzed. For the 15-minute no-drug session, the number of nose pokes in the previously active and inactive holes was analyzed. The percentage changes in poking rates (nose pokes per minute) between the first drug session and the following no-drug period were calculated. Sphericity assumed modeling with Greenhouse-Geisser and Huynh-Feldt adjustments was applied [31].

## Results

### Experiment 1

#### Total number of infusions during acquisition

Phenotype significantly affected the total number of infusions during the acquisition phase (*F*_1,29 _= 25.481, *P *< 0.001). *Post hoc *analysis showed that bHR males and females had a greater number of infusions during the FR1 and FR2 schedules than their bLR counterparts (*F*_1,29 _= 11.730, *P *= 0.001, and F_1,29 _= 11.730, *P *= 0.002, respectively; data not shown). Greater cocaine intake prior to the PR schedule did not enhance the BP; in fact, the only correlation identified was for bHR females, in which greater cocaine intake during acquisition was negatively correlated with the BP at the 0.5 mg/kg/infusion dose (*r *= -0.719, *P *= 0.013).

#### Impact of bHR-bLR phenotype and sex on breaking point

**Figure 1:**
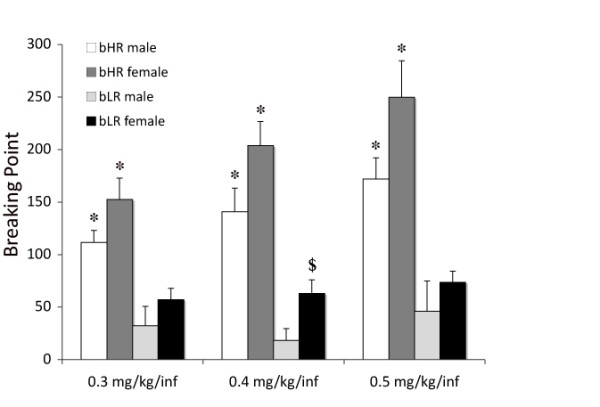
**Breaking points (BPs) on a progressive ratio (PR) schedule of reinforcement for bred High Responder (bHR) and bred Low Responder (bLR) males and females at three different doses of cocaine (Experiment 1)**. *P < 0.05, significant effect of phenotype within sex at that dose; ^$^*P *< 0.05, significant effect of sex within phenotype at that dose.

When animals were tested using a PR schedule of reinforcement to determine the BP for cocaine self-administration, there were significant main effects of phenotype (bHR > bLR, *F*_1,29 _= 43.475; *P *< 0.001), sex (female > male, *F*_1,29 _= 6.720; *P *= 0.015) and dose (*F*_2,59 _= 5.301, *P *= 0.008) (see Figure 1). *Post hoc *analyses were conducted to examine the effect of phenotype within sex and dose, and we found that bHR males had higher BPs than bLR males at all doses (low to high dose: *P *= 0.003, *P *< 0.001, *P *= 0.001), and bHR females had higher BPs than bLR females at all three doses (low to high dose: *P *= 0.001, *P *< 0.001, *P *< 0.001). *Post hoc *analyses also revealed that within dose and phenotype, bLR females had higher BPs than bLR males at the 0.4 mg/kg/infusion dose (*P *= 0.05). While a main effect of cocaine dose on BP was also found (*F*_2,59 _= 5.301, *P *= 0.008), *post hoc *analyses indicated that the dose effect was only significant for the bHR females with the BP being higher at the highest dose of cocaine (0.5 mg/kg/infusion) compared with the lowest dose of cocaine (0.3 mg/kg/infusion) (*P *= 0.012).

### Experiment 2

#### Total number of infusions during acquisition of cocaine self-administration

Phenotype significantly affected cocaine intake during acquisition (*F*_1,37 _= 6.304, *P *= 0.017), with bHR males administering a greater number of infusions during FR1 and FR2 than their bLR counterparts (*F*_1,37 _= 5.385, *P *= 0.026). The effect was not significant in females (data not shown). BP was not correlated with the number of infusions during FR1 and FR2 for any of the groups at any of the cocaine doses (data not shown).

#### Impact of bHR-bLR phenotype and sex on breaking point using an expanded dose range

**Figure 2:**
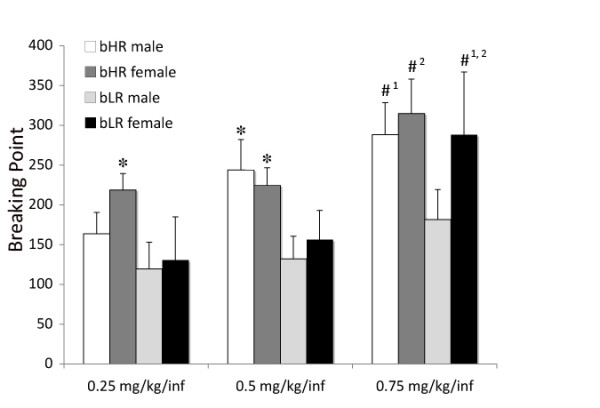
**BPs on a PR schedule of reinforcement for bHR and bLR males and females using an expanded range of doses of cocaine (Experiment 2)**. *P < 0.05, significant effect of phenotype within sex at that dose. ^#^*P *< 0.05, statistically significantly different from the lowest dose (^#1^) or the middle dose (^#2^) within sex and phenotype.

When the dose range for BP was expanded compared with Experiment 1, there were no significant main effects, but there were significant interaction effects for day and sex (*F*_4,132 _= 4.213, *P *= 0.003); day, sex and phenotype (*F*_4,132 _= 2.614, *P *= 0.038); and dose and day (*F*_8,264 _= 3.371, *P *= 0.012). *Post hoc *analyses indicated that at 0.25 mg/kg/infusion and 0.5 mg/kg/infusion, bHR females had a higher BP than bLR females (*P *= 0.015 and *P *= 0.044, respectively), and at 0.5 mg/kg/infusion, bHR males had a higher BP than bLR males (*P *= 0.026) (see Figure 2). bLR females showed a significant increase in BP between the 0.25 and 0.75 mg/kg/infusion doses (*P *< 0.005) and between the 0.5 and 0.75 mg/kg/infusion doses (*P *< 0.005). bHR females demonstrated increased BP between the 0.5 and 0.75 mg/kg/infusion doses (*P *< 0.05). bHR males showed a significant increase in BP between the 0.25 and 0.75 mg/kg/infusion doses (*P *< 0.05) (see Figure 2). Thus, BP was highest at the 0.75 mg/kg/infusion dose for all groups except the bLR males, which failed to show an increase in BP relative to the two lower doses.

#### Impact of bHR-bLR phenotype and sex differences on responding on an FR5 schedule of reinforcement and during periods with no reinforcement

**Figure 3:**
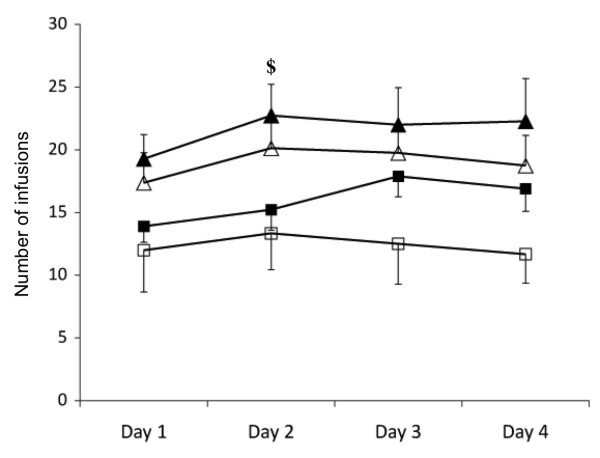
**The number of infusions self-administered during the 40-minute drug-available sessions using a fixed ratio 5 schedule of reinforcement**. ^$^*P *< 0.05, significant effect of sex within phenotype for that day. Closed squares, bHR males; closed triangles, bHR females; open squares, bLR males; open triangles, bLR females.

Animals were tested in two 40-minute drug-available sessions (a FR5 schedule with a 40-second timeout after infusions) that were separated by one 15-minute no-drug period (when responses in the holes were recorded but not reinforced). A significant effect of sex was found on cocaine intake (*F*_1,28 _= 5.940, *P *= 0.021), with female rats, regardless of phenotype, self-administering a greater number of cocaine infusions than males (see Figure 3). This greater drug intake was driven primarily by higher cocaine intake by bHR females compared with bHR males on the second day of testing (*P *= 0.02), with a similar trend observed on the first day (*P *= 0.06).

**Figure 4:**
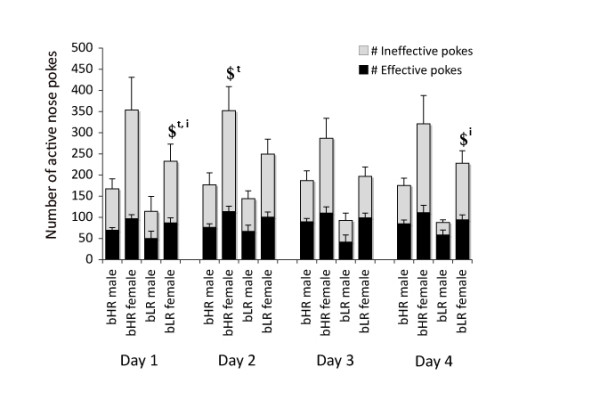
**The total number of nose pokes in the active hole during the drug-available sessions separated by effective nose pokes (those that count toward an infusion of cocaine) and ineffective nose pokes (nose pokes during the 40-second timeout)**. ^$^*P *< 0.05, significant effect of sex within phenotype for that day for total active pokes ($^t^) and ineffective active pokes ($^i^).

A significant main effect of sex was found for the total number of nose pokes in the active hole (*F*_1,28 _= 6.023, *P *= 0.021) during the drug-available session during the FR5 sessions (see Figure 4). bLR females demonstrated more active nose pokes than bLR males on day 1 (*P *= 0.039), and bHR females demonstrated more active nose pokes than bHR males on day 2 (*P *= 0.041). A significant main effect of sex was also found for the number of ineffective nose pokes (nose pokes during the 40-second timeout after each infusion) in the active hole (*F*_1,28 _= 6.381, *P *= 0.017), with bLR females poking more ineffectively on day 1 (*P *= 0.042) and day 4 (*P *= 0.039).

**Figure 5:**
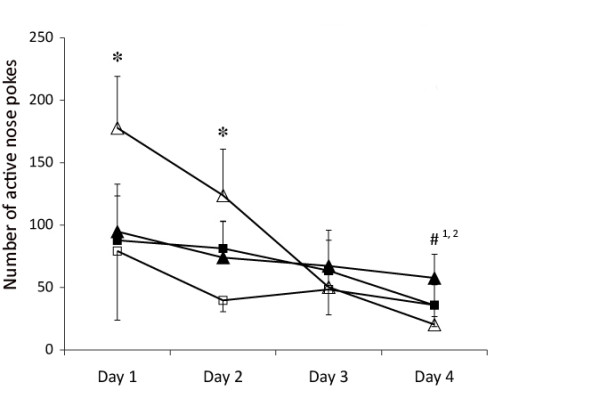
**The number of nose pokes in the active hole during the 15-minute no-drug period, at which time pokes were not reinforced**. *P < 0.05, significant effect of phenotype within sex for that day. ^#^*P *< 0.05, statistically significantly different from day 1 (#^1^) or day 2 (#^2^) for bLR females. Closed squares, bHR males; closed triangles, bHR females; open squares, bLR males; open triangles, bLR females.

The number of nose pokes in the active hole during the 15-minute no-drug periods between the FR5 drug-available sessions showed significant interaction effects between day, sex and phenotype (*F*_3,269 _= 4.420, *P *= 0.008) (see Figure 5). On days 1 and 2, bLR females demonstrated more pokes in the active hole during the 15-minute no-drug period compared with bHR females (*F*_1,23 _= 5.413, *P *= 0.029, and *F*_1,23 _= 4.754, *P *= 0.040, respectively). Additionally, bLR females poked significantly less often in the active hole during this no-drug period on day 4 compared with days 1 and 2 (*P *< 0.01).

#### Percentage change in active hole nose pokes

There was a significant interaction between sex and phenotype on the change in poking rates in the active hole between the drug and no-drug sessions (*F*_1,27 _= 5.76, *P *= 0.028), and there was a sex, phenotype and day interaction (*F*_3,27 _= 3.262, *P *= 0.032). As illustrated in Table 1, on the first day of FR5, bLR females increased their poking rate by an average of 300% (*F*_1,58 _= 8.923, *P *= 0.004) during the no-drug session compared with the drug-available session (that is, compared to 100%) bLR females also showed a significantly greater percentage change in poking rates than bLR males (*F*_1,27 _= 7.013, *P *= 0.013) and bHR females (*F*_1,27 _= 9.647, *P *= 0.004). This increase in poking rate in the active hole decreased during subsequent days (day 1 vs. day 3, *P *= 0.03; day 1 vs. day 4, *P *= 0.005), with poking rate significantly decreasing to 20% of the drug-available session on day 4 (*F*_1,58 _= 4.395, *P *= 0.04). On day 2, bLR females still had a higher percentage change than their bHR counterparts (*F*_1,27 _= 6.966, *P *= 0.014). The other groups showed no change in poking rates between the drug and no-drug sessions.

**Table 1:** Change in the number of active nose pokes per minute between the first drug-available and no-drug sessions expressed as percentage changes from the drug-available session^a^

Rat type	Day 1	Day 2	Day 3	Day 4
bHR male	112.82 (33.0)	131.70 (33.3)	109.43 (46.5)	83.08 (46.4)
bHR female	72.17 (24.2)	59.54 (16.6)	60.77 (9.24)	82.00 (34.8)
bLR male	46.30 (24.5)	87.53 (10.3)	100.98 (46.7)	83.62 (51.8)
bLR female	290.85 (102.0)^b,c,d^	142.93 (37.5)^c^	88.03 (33.1)^e^	21.39 (5.0)^e^

^a^bHR, bred High Responder; bLR, bred Low Responder; data are expressed as means ± SEM; ^b^*P *< 0.05, effect of sex within phenotype for that day; ^c^*P *< 0.05, significantly different from 100%, *P *< 0.05; ^d^*P *< 0.05, effect of phenotype within sex for that day; ^e^*P *< 0.05, significantly different from Day 1.

We did not identify any significant effects of estrous cycle on any behaviors measured for either phenotype (data not shown). In Experiments 1 and 2, 15 of 20 and 14 of 19 of the females, respectively, exhibited regular estrous cycles for the duration of the study.

## Discussion

In the present experiments, we investigated sex differences and the effect of selective breeding for novelty reactivity on the motivation to self-administer cocaine and for responding in the absence of reinforcement. We report that bHR males and females exhibited higher motivation to self-administer cocaine compared with bLR rats. There is also an underlying sex difference in the motivation to self-administer cocaine regardless of phenotype, with females showing greater motivation than males. Motivation for cocaine increased with dose, so that all animals except bLR males demonstrated significantly greater BPs at the highest dose tested (0.75 mg/kg/infusion) compared with lower doses. Thus, motivation contributes to the increased cocaine-taking seen in bHR females and is likely affected by both sex and phenotype.

### Impact of the bHR-bLR phenotype on motivation for cocaine

Prior research conducted at our laboratory demonstrated that bHR males and females acquire cocaine self-administration behavior significantly faster than bLR males and females, and bHR female rats self-administer greater amounts of cocaine than bHR males and bLR males and females [5]. The current study aimed to determine whether the higher drug intake previously seen in bHR females is due to higher motivation for the drug. We found that, in general, bHR rats exhibited higher BPs than bLR rats, indicating an increased motivation to take cocaine in the bHR versus bLR animals.

The mesolimbic dopamine system, specifically dopaminergic projections from the ventral tegmental area (VTA) to the nucleus accumbens (NAc), has been studied extensively for its role in mediating the rewarding effects of drugs of abuse and may be responsible for mediating the differences in motivation seen in the bHR and bLR animals. Indeed, bHR and bLR animals exhibit differences in their dopaminergic system, with bHR animals exhibiting differences in dopamine (DA) receptors and sensitivity [12]. Studies conducted with outbred HR and LR animals have shown similar findings, with HR animals exhibiting increased responsivity to DA [32-34], higher basal DA levels in the NAc [32] and more persistent elevation in DA cell firing in the VTA compared with LR animals [35], a neuroadaptation that appears to be critical for the development of addictive behaviors [36,37]. These differences in the dopaminergic reward system may play a role in the enhanced motivation for cocaine that we see in bHR animals.

Normally, within the dose range used in this study, the amount of cocaine administered increases with dose [24]. Interestingly, bLR males did not exhibit amplified motivation to take cocaine at the highest dose, in contrast to significant increases in motivation demonstrated by the other groups, including bLR females. It appears that while the bLR phenotype is "protective" to a certain degree by reducing the motivation for cocaine, this protection is limited in females, and ultimately sex overrides behavioral phenotype in this situation, resulting in increased response for cocaine at a high dose (0.75 mg/kg/infusion).

The level of motivation to self-administer cocaine can be influenced by prior cocaine intake during self-administration training (for review, see [38]). For example, a level of 20 mg/kg/day during initial exposure results in an escalation of subsequent BPs, whereas an intake of 60 or even 100 mg/kg/day results in stable responding on a PR schedule [39]. In the experiments presented here, the 5- and 7-day FR "training" period that preceded PR testing resulted in bHR rats' receiving more cocaine than bLR rats, which could have affected the animals' motivation for cocaine and subsequent BPs. However, the intake differences in the current study were significantly less than those reported by Morgan *et al. *[39], where they resulted in significant differences in BP, with intake differences remaining within 3 mg/kg/day.

### Impact of sex on motivation for cocaine

Females, in addition to acquiring cocaine self-administration faster [5], also have higher motivation than males to take cocaine. Thus, females may be more sensitive than males to the reinforcing effects of cocaine. These data confirm results in other reports that females are willing to work harder than males for the same dose of cocaine [22,25,40].

While females generally exhibit higher motivation for cocaine than males, all differences in motivation to self-administer cocaine were obscured at the highest dose (0.75 mg/kg/infusion). This finding is similar to what we have seen before, in that drug-taking differences are often exhibited only at lower cocaine doses [23]. Motivation to self-administer cocaine often increases with escalating doses; thus, drug-taking differences are often obscured when high cocaine doses are administered, as all animals tend to exhibit elevated motivation for the drug at higher doses. It is often only at the lowest doses that subtle but significant differences emerge [23,26].

It is interesting to note, however, that while the bLR females demonstrated a significant increase in motivation to self-administer cocaine at the highest dose, the bLR males did not. It is possible that the bLR females are more responsive than bLR males to the effects of cocaine. The effect of sex on motivation for cocaine may override the bLR phenotype at higher doses for the females, resulting in increased motivation for cocaine compared with bLR males.

Neurobiological differences between LR and HR and between bHR and bLR rats have been described for male rats; however, several of the brain regions where differences have been found are known to be sensitive to estradiol in females. For example, bHR males have a greater proportion of dopamine D2 (high) receptors in the striatum [12], and our laboratory has shown that dopamine release in the striatum is augmented by estradiol in females [41]. In addition, corticotropin-releasing hormone (CRH) mRNA levels in the hypothalamic paraventricular nucleus (PVN) are lower in LR rats [11]. Estrogen receptors affect activation of CRH neurons in the PVN [42], and estradiol in the PVN also modulates the responsivity of the stress axis [43]. It is possible, therefore, that estradiol in the bLR females augments the rewarding effect of cocaine, but only at higher doses.

On the other hand, it is also possible that the brains of the bLR males are simply less responsive to the effects of the cocaine. Indeed, Flagel *et al. *[12] found differences in dopaminergic circuitry in bLR males compared with bHR males, and we have seen that bLR males do not exhibit as robust a behavioral response to cocaine sensitization as do bHR males [44].

Gonadal hormones have been shown to play a significant role in the sex differences that exist with regard to drug-taking behavior [21,22,25]. We did not, however, find an effect of estrous cycle on the motivation to take cocaine in the experiments presented here, even though the majority of females continued to exhibit regular estrous cycles for the duration of the experiment. We previously reported a similar lack of effect of the estrous cycle on the acquisition of cocaine-taking behavior [5], which is likely due to the large number of females that exhibited aberrant cycles or stopped cycling altogether as a result of cocaine exposure. Lynch *et al. *[27] also reported irregular estrous cycles during cocaine self-administration in female rats. It is important to note that the previous studies reporting the effects of endogenous estrous cycles on BPs differed from ours in a number of ways [25]. For example, the animals in previous studies were reinforced with a much higher cocaine dose during self-administration training and PR testing (0.6 mg/infusion) than the dose that we used. The animals in the other studies also self-administered cocaine for a shorter period and were housed in self-administration boxes for the duration of the study. These are important methodological differences that could potentially affect the results.

Additionally, our finding of a lack of effect of the estrous cycle may be due, at least in part, to the variation in circulating hormone levels between females at the time of testing. We have previously seen clear effects of estradiol on cocaine self-administration when administered to OVX females [23,26]. In these prior studies, however, the time of behavioral testing was tightly regulated with regard to the exogenously administered hormone. Variation between females in circulating hormone levels at the time of testing, independent of lavage readings, could add to the variation seen in motivation, which could obscure differences that would otherwise be identified during *post hoc *analysis.

Thus, the sex differences in cocaine self-administration may be exacerbated by, but do not appear to require, high levels of circulating gonadal hormones. Indeed, we have shown previously that OVX females that were not given estradiol replacement exhibited higher levels of self-administration than males, confirming that circulating hormones are not necessary to maintain a female's increased drug abuse liability [26].

### Impact of phenotype and sex on continuing to response without reinforcement

In recent years, researchers have been attempting to more closely model human addiction by adding tests based on the criteria in the *Diagnostic and Statistical Manual of Mental Disorders*, *Fourth Edition *[45], for substance dependence, such as persisting to respond to a drug in the absence of reinforcement or in the presence of a punishment [6,46]. Animals that are unable to refrain from responding when the drug is not received are said to demonstrate increased drug-seeking, a marker of addiction.

Thus, in Experiment 2, we added a test to examine the continuation of responding in the absence of reinforcement. During the first two 15-minute drug-free sessions, bLR females responded significantly more often than the bLR males; there were no differences between the bHR groups. To compare rates of responding during the drug-free and drug-available sessions, we calculated the number of pokes per minute during the 15-minute drug-free session and expressed that number as a percentage change from the poking rate during the drug-available session. In this part of the experiment, it was clear that the bLR females exhibited a significant increase in rates of responding during the non-reinforced (that is, drug-free) period compared with the period when the drug was available. According to these data, the bLR females demonstrated increased drug-seeking in the absence of reinforcement. However, analysis of the poking rate on subsequent days demonstrated that the bLR females were the only group that changed its rate of poking during the drug-free period in successive sessions and actually decreased its rates of responding during periods of no reinforcement on subsequent days. This change in poking rate was specific for the drug-free period, since poking rate during the drug-available session did not change. Interestingly, the other groups continued to respond at about the same rate as during the preceding drug-available period, showing no sign of learning that nose poking was not being reinforced during the drug-free session.

## Summary and conclusion

The experiments presented here are the first to evaluate how the selectively bred, novelty-seeking phenotype as well as the sex of an individual affect motivation to self-administer cocaine. An individual's sex continues to increase drug abuse liability. We found underlying sex differences in motivation to take cocaine, with females having higher BPs than males, regardless of phenotype, demonstrating that these factors are dissociable. Additionally, reactivity to novelty affected motivation to take cocaine, as bHR animals had higher BPs than bLR animals at most of the doses tested. At the highest dose (0.75 mg/kg/infusion), all phenotype and sex differences disappeared, as all groups (except for bLR males) demonstrated significantly higher BPs than they did at the lower doses.

## Competing interests

The authors declare that they have no competing interests.

## Authors' contributions

JC participated in the design of the experiment, acquired all data, assisted in the input of data for analysis, participated in the interpretation of the data and drafted the manuscript. BG participated in the design of the experiment, assisted in gathering and inputting the data for analysis and assisted with the editing the manuscript. CW assisted in the input of data for analysis, conducted statistical analyses and assisted in the drafting of the manuscript. SC prepared and coordinated selectively bred animals prior to the experimental procedures and assisted in the editing of the manuscript. HA conceived of the bHR/bLR lines and contributed to the editing of the manuscript. JB conceived of the project, participated in its design and assisted in drafting and editing the manuscript. All authors read and approved the final manuscript.
